# Two Cases of *ASXL3*‐Related Bainbridge–Ropers Syndrome: Clinical and Genetic Evaluation

**DOI:** 10.1155/crig/9304690

**Published:** 2026-09-03

**Authors:** Şirin Sedef Baş, Burcu Yeter, Nursel H. Elcioglu

**Affiliations:** ^1^ Department of Pediatric Genetics, Marmara University Medical School, Istanbul, Turkey; ^2^ Department of Pediatric Genetics, Umraniye Training and Research Hospital, Istanbul, Turkey, ueh.gov.tr

**Keywords:** *ASXL3*, Bainbridge–Ropers syndrome, developmental delay

## Abstract

*ASXL3*‐related disorder, also known as Bainbridge–Ropers syndrome (BRPS), is a neurodevelopmental condition first described by Bainbridge et al. and characterized by delayed psychomotor development, learning difficulties, characteristic craniofacial features, hypotonia, behavioral problems, and feeding problems. It is associated with heterozygous truncating pathogenic variants in the *ASXL3* gene, located at chromosomal region 18q12.1. In this study, exome sequencing was performed in a 7‐month‐old male and a 4‐year‐old female patient who presented with unexplained developmental delay. De novo truncating variants in the *ASXL3* gene were identified in both patients. Genetic analysis revealed that Patient 1 had a canonical splice‐site likely pathogenic variant in the *ASXL3* gene c.3039 + 1G > *A*. Patient 2 had a pathogenic variant in the *ASXL3* gene c.1864_1867dup; p.Thr623Metfs^∗^25. We compared the patients’ physical examination findings, clinical characteristics, and genetic results with those reported in the literature. This case report emphasizes the phenotypic spectrum of this disorder and describes a variant that, to our knowledge, has not been previously reported in the literature.

## 1. Introduction

Bainbridge–Ropers syndrome (BRPS; MIM# 615485) is a rare neurodevelopmental disorder. BRPS characterized by learning difficulties, behavioral abnormalities, delayed psychomotor development, severe speech and language impairment or absence of speech, hypotonia, feeding difficulties, and craniofacial features. Characteristic craniofacial features include prominent forehead, downslanting palpebral fissures, a high‐arched palate, full and everted lower lip, arched eyebrows, a low columella, broad nasal tip, and anteverted nares [1]. The disorder was first described in 2013 by Bainbridge et al., who identified de novo truncating variants in the *ASXL3* gene in four unrelated individuals [2]. The Additional Sex Combs‐Like (*ASXL*) gene family comprises three members: *ASXL1*, *ASXL2*, and *ASXL3*. These three genes play important roles in embryonic development and in the interpretation of post‐translational histone modifications [3]. Germline pathogenic variants in *ASXL1* are associated with Bohring–Opitz syndrome, while variants in *ASXL2* cause Shashi–Pena syndrome [4]. Due to its rarity and phenotypic variability, diagnosis may be challenging. BRPS shows considerable phenotypic variability, even among patients with similar variants. Genotype–phenotype correlations remain incompletely understood. Given the limited number of reported cases of BRPS and the identification of a potentially novel variant in one of our patients, we present these two cases. In this study, we aimed to present the clinical and genetic features of two patients with *ASXL3*‐related BRPS and to contribute to the expanding phenotypic spectrum of this condition.

## 2. Materials and Methods

Exome sequencing was performed on two pediatric patients who were admitted to the Department of Pediatric Genetics due to unexplained developmental delay. Chromosome analysis and microarray testing performed on peripheral blood samples of the patients did not reveal any clinically significant findings. The genetic investigations of the two patients were performed at the same center but in different years. As available gene panels continuously evolve over time, different testing approaches were used despite the similarity of the clinical presentations.

Clinical exome sequencing (CES) analysis was performed in Patient 1. The laboratory workflow briefly involves amplification of the disease‐associated gene regions by polymerase chain reaction (PCR) and sequencing of these regions using next‐generation sequencing technology. For this purpose, the Clinical Exome Solution by SOPHiA Genetics kit was used. Sequencing reactions were performed using the Illumina NextSeq system and compatible reagent kits. Raw data were analyzed using the SOPHiA DDM data analysis platform. Alignment and variant calling were performed against the hg19 human genome reference using Pepper, a proprietary core algorithm of SOPHiA Genetics. Variant annotation was carried out using the MOKA software by SOPHiA Genetics. In addition to variant effects within the gene (missense, stop‐gain, etc.), various population frequency databases (1000G, ESP, ExAC, and gnomAD), frequency data, predictive algorithms (SIFT and PolyPhen), and information on the variant’s potential pathogenicity were evaluated. Next‐generation sequencing was conducted using Illumina sequencing‐by‐synthesis (SBS) technology, with a minimum read depth of 50 × per nucleotide. Variant interpretation was based on the guidelines of the American College of Medical Genetics and Genomics (ACMG) 2015. Since comprehensive genome and exome reference databases specific to the Turkish population are limited, the 1000 Genomes Project, dbSNP, and ExAC databases were used as control populations. The coverage obtained for the target regions was 99.49% at 20×, 99.16% at 25×, 98.66% at 30×, 94.89% at 50×, 77.26% at 100×, and 45.39% at 200×.

Whole exome sequencing (WES) analysis was performed in Patient 2. The laboratory workflow briefly involves amplification of the disease‐associated gene regions by PCR and sequencing of these regions using next‐generation sequencing technology. For this purpose, the Twist Human Core Exome v2 kit was used. Sequencing reactions were performed using the Illumina NovaSeq system and compatible reagent kits. Bioinformatic analyses were carried out using SOPHiA _v5. All steps, from raw data processing to variant detection, were performed using the SOPHiA _v5 software and its algorithms. Raw data were aligned to the hg19 human genome reference. As a result of the alignment, BAM files were generated. Variants (SNP/INDEL) were reported in VCF format. Coverage information was also calculated during the analysis process by the SOPHiA _v5 software. Annotation of the obtained VCF files was performed using the Ensembl VEP software. Transcript prioritization was conducted using the “pick order” option of VEP in the following order: “refseq, mane, canonical, cdsrank, biotype, appris, length,” and the predicted transcripts were listed accordingly. Variants with a variant allele fraction below 25% and read depth below 20 were excluded from the analysis.

## 3. Results

Patient 1, a male, 7 months infant, presented with neuromotor developmental delay.

### 3.1. Prenatal/Perinatal History

There were no significant prenatal abnormalities. The patient was born to a G1P1 mother by cesarean section, with a birth weight of 3200 g (50th percentile), a length of 50 cm (58th percentile), and a head circumference of 35 cm (62nd percentile). The parents were nonconsanguineous, and there was no known family history of genetic or neurological disorders. At 20 days of age, the patient was admitted to the neonatal intensive care unit (NICU) because of jaundice and respiratory distress.

### 3.2. Physical Examination

Physical examination included postnatal growth failure, delays in neuromotor milestones, poor head control, and hypotonia. Dysmorphic features included a depressed nasal bridge, frontal bossing, hypertelorism, thin and highly arched eyebrows, mild proptosis, and full lips.

### 3.3. Neurodevelopment and Other Clinical Features

Due to failure to thrive and recurrent vomiting since birth, the patient underwent evaluations at multiple centers. Neuromotor developmental delay and vomiting prompted further assessment by pediatric metabolic specialists.

### 3.4. Imaging and Other Investigations

Electroencephalography (EEG), cranial computed tomography (CT), and magnetic resonance imaging (MRI) examinations were requested. The cranial CT was unremarkable, while the cranial MRI demonstrated diffuse, symmetric white matter signal abnormalities. EEG findings were normal. Hearing tests, thyroid ultrasound, and abdominal ultrasound were all within normal limits. Echocardiography revealed the presence of a Patent Foramen Ovale (PFO). Ophthalmological examination was reported as normal.

Exome sequencing identified a heterozygous canonical splice‐site pathogenic variant in the *ASXL3* gene NM_030632.3:c.3039+1G > *A*. When evaluated according to ACMG criteria, the variant was classified as likely pathogenic (PVS1_Strong, PM2, PP5, and PM6). Loss of function is a known mechanism of the disease. The variant is located in exon 11 of a 12‐exon transcript, and exon skipping disrupts reading frame. This variant is predicted not to undergo nonsense‐mediated decay (NMD) but as pathogenic variants have been reported distal to this position, loss of function of the resulting protein may still be inferred (PVS1_Strong). This variant is absent in the general population (PM2). It has been reported as pathogenic by ClinVar (Variation ID: 622811). Reputable source reports variant as pathogenic, but the evidence is not available to the laboratory to perform an independent evaluation (PP5). Sanger sequencing of the parents showed no variant, confirming the variant as de novo (PM6). The patient was subsequently diagnosed with BRPS and enrolled in follow‐up.

### 3.5. Follow‐Up Evaluation at 2 Years of Age

**FIGURE 1:**
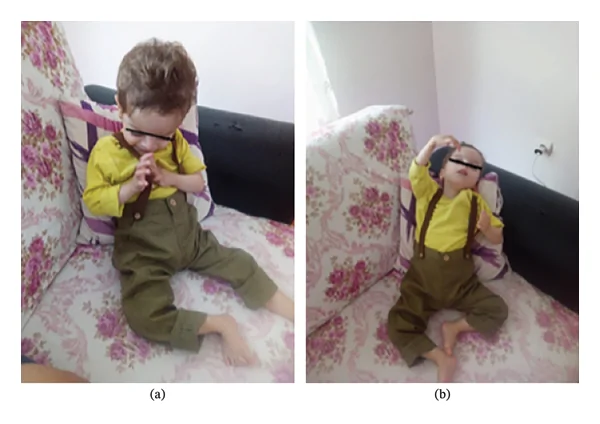
Patient 1: (a) demonstrating frontal bossing and (b) highlighting postnatal growth failure and poor head control.

**FIGURE 2:**
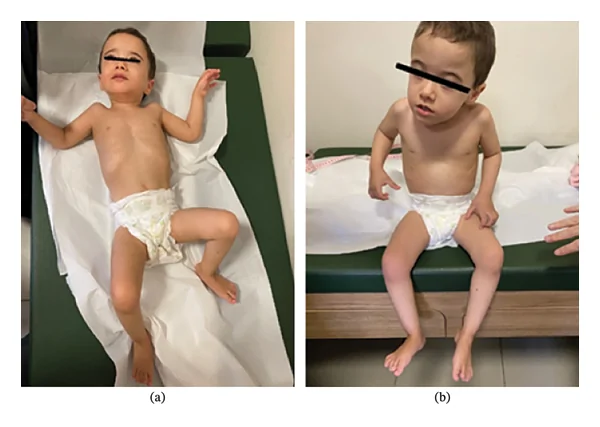
Patient 2: (a) revealing a prominent metopic suture, hypertelorism, and short nose and (b) demonstrating pectus excavatum, overriding toes, and clinodactyly of the fourth and fifth toes.

The patient was re‐evaluated at 2 years of age. There was no history of seizures. The patient has been receiving physiotherapy for 1 year and began to roll over following the initiation of therapy. At the age of 2 years, the patient’s anthropometric measurements were as follows: weight 7 kg (−5.7 SDS), height 81 cm (−2.6 SDS), and head circumference 45 cm (−2.97 SDS). Developmental milestones were significantly delayed: head control was achieved at 12 months of age; the patient was able to sit with support but has not yet achieved independent sitting. He was unable to walk, and speech has not developed. The first tooth erupted at 14 months of age. (see Figures 1 and 2)

Patient 2, a 4‐year‐old female patient, was referred due to neuromotor developmental delay.

### 3.6. Prenatal/Perinatal History

The patient was born to a G3P2A2 mother via cesarean section at 33 + 4/7 weeks of gestation. Birth weight was 1100 g (−2.43 SDS), and length was 39 cm (−1.67 SDS). The patient was born from a twin pregnancy; the cotwin is healthy. There is no consanguinity between the parents and no known family history of genetic or neurological disorders. The patient was monitored in the NICU for 45 days due to prematurity and respiratory distress, requiring mechanical ventilation.

### 3.7. Physical Examination

Anthropometric measurements at the time of evaluation were as follows: weight 8 kg (−5.78 SDS), length 80 cm (−5.12 SDS), and head circumference 45.5 cm (−3.17 SDS). The anterior fontanelle was closed at 2.5 years of age. Physical examination revealed a prominent metopic suture, epicanthus, hypertelorism, upslanting palpebral fissures, depressed nasal bridge, short nose, pectus excavatum, overriding toes, clinodactyly of the fourth and fifth toes, mild central hypotonia, and postural scoliosis.

### 3.8. Neurodevelopment and Other Clinical Features

Developmental milestones: Head control was achieved on time. The patient was able to sit with support at 18 months and without support at 24 months. She is not yet able to walk independently but is able to take steps with assistance from her parents. Speech is absent; she produces only nonspecific vocalizations and syllable repetitions. The first tooth erupted at 12 months of age. Due to prematurity, the patient underwent retinopathy of prematurity (ROP) screening. The most recent ophthalmological examination showed no evidence of ROP; however, follow‐up with ophthalmology continues due to mild strabismus. Newborn screening revealed elevated TSH levels, and the patient is currently on levothyroxine treatment under pediatric endocrinology follow‐up. Echocardiographic evaluations performed both during the NICU stay and at 1 year of age were normal. Given prematurity and delays in neuromotor milestones, the patient is under regular follow‐up with pediatric neurology and pediatric metabolic specialists. Due to gastroesophageal reflux and inadequate weight gain, the patient was formula fed until the age of two and was followed by pediatric gastroenterology. Her reflux symptoms resolved after this period, and follow‐up was discontinued.

### 3.9. Imaging and Other Investigations

Brain diffusion MRI was reported as normal, and metabolic investigations yielded no significant findings. EEG findings revealed an age‐appropriate normal bioelectrical background activity, with evidence of a paroxysmal irregularity characterized by sharp and sharply contoured slow waves in the 4–7 Hz frequency range. There is no history of seizures. Chromosomal analysis showed a normal female karyotype (46, XX). Chromosomal microarray analysis identified a heterozygous 1.9‐Mb gain at 10p15.3p15.2 (arr[GRCh37] 10p15.3p15.2(1,524,409_3,439,835)x3). The same region showed a 1.83‐Mb gain in the father’s microarray, overlapping with the patient’s duplication, and the array result was not significant. WES revealed a heterozygous frameshift variant in exon 11 *ASXL3* NM_030632.2: c.1864_1867dup; p.Thr623Metfs × 25. When evaluated according to ACMG criteria, the variant was classified as pathogenic (PVS1, PM2, and PM6). The c.1864_1867dup (p.Thr623Metfs^∗^25) variant is a frameshift variant predicted to introduce a premature termination codon in exon 11 of the 12‐exon ASXL3 transcript. The resulting transcript is expected to undergo nonsense‐mediated mRNA decay. Loss of function is the established disease mechanism for ASXL3‐related BRPS (PVS1). This variant is absent in the general population (PM2). Parental Sanger sequencing of *ASXL3* was carried out to verify the pathogenic variant and establish its de novo origin (PM6).

Written informed consent was obtained from the participants’ parents for the publication of this case report and the accompanying clinical photographs (see Tables 1 and 2).

**TABLE 1:** Perinatal and clinical examination findings.

	Case 1	Case 2
Antenatal history	—	Twin pregnancy

Gestational age at birth	38 weeks	33 + 4/7 weeks

Birth weight/length/HC	3200 g (50th percentile)	1100 g (–2.4 SDS)
	Length: 50 cm (58th percentile)	Length: 39 cm (–1.67 SDS)
	HC: 35 cm (62th percentile)	HC: Unknown

Sex	Male	Female

Age at diagnosis	7 months	4 years

Current age	2 years	4 years

Anthropometrics at presentation	Weight: 7 kg (–5.7 SDS)	Weight: 8 kg (–5.78 SDS)
	Height: 81 cm (–2.6 SDS)	Height: 80 cm (–5.12 SDS)
	HC: 45 cm (–2.97 SDS)	HC: 45.5 cm (–3.17 SDS)

Physical examination findings		
Microcephaly	+ (Secondary)	+
Prominent forehead	+	+
Hypertelorism	+	+
Strabismus	–	+
Arched eyebrows	+	–
Prominent nasal bridge	+	–
Broad nasal root	–	+
Low columella	+	–
Short nasal bone	–	+
Anteverted nares	–	+
High‐arched palate	–	+
Pectus excavatum	–	+
Scoliosis	–	+

**TABLE 2:** Clinical and genetic findings.

	Case 1	Case 2
Neurological findings		
Brain MRI	Diffuse symmetric signal increase in the white matter	Diffusion MR is normal
Seizure history	–	–
EEG result	Normal	A paroxysmal irregularity characterized by sharp waves and sharply contoured slow waves
Developmental delay	+	+
Hypotonia	+	+
Speech	–	–
Feeding problems or GERD	Vomiting	GERD
Poor sucking/swallowing	+	–
Growth retardation	+	+
Hand‐flapping movements	–	–
Ophthalmological examination	–	Strabismus
Hearing test	Normal	Normal
Additional finding	–	–
Variant	*ASXL3* (NM_030632.3) c.3039+1G > *A* intronic	*ASXL3* (NM_030632.2) exon 11 c.1864_1867dup (p.Thr623Metfs^∗^25)
Type of variant	Splice	Frameshift
Inheritance of the variant	De novo	De novo
Previously reported to our knowledge	+	–

## 4. Discussion

The *ASXL* gene family—comprising *ASXL1* (located at 20q11.21), *ASXL2* (at 2p23.3), and *ASXL3* (at 18q12.1)—represents the human orthologs of the additional sex combs (*Asx*) gene in *Drosophila*, which encodes a key regulator of the Polycomb‐group (PcG) repressor complex (PRC) [5]. The *ASXL3* protein is composed of 2248 amino acids, with most of the coding sequence concentrated in the final two exons—exon 11 and exon 12 [3]. This genomic organization contributes to the variability observed in the coding potential across the *ASXL* gene family. The majority of the identified variants are de novo truncating variants, which are predicted to result in loss of function [3, 6]. In this study, we identified two Turkish patients with BRPS. Most of the previously reported variants were de novo, with parental genetic testing confirming this in the majority of cases; however, there have also been genetically confirmed inherited cases [7, 8].

Both patients were referred to our clinic because of delayed neuromotor development. In our patients, other prominent clinical features included feeding difficulties, hypotonia, and the absence of speech. Microcephaly, a prominent forehead, and hypertelorism were common findings on physical examination. Bainbridge et al. [2] described four unrelated individuals carrying de novo heterozygous truncating variants in *ASXL3*, all exhibiting comparable clinical features such as severe feeding difficulties, failure to thrive, and neurological abnormalities associated with marked developmental delay.

In the literature, speech absence or delay has been reported in 95% of the cases [3]. In our patients, neither patient had developed speech. According to previous reports, 88% of the cases exhibited hypotonia [3]. Balasubramanian et al. [9] reported hypotonia in all 12 patients. Similarly, the presence of hypotonia in both of our cases was noteworthy, consistent with previous reports.

In the study by Kuechler et al. [10], which included six patients with BRPS, feeding difficulties were reported in all individuals. According to previous reports, 76% of the cases exhibited feeding difficulties, which were most severe in infancy [3]. Consistent with these findings, our first patient experienced vomiting, while the second was diagnosed with gastroesophageal reflux disease (GERD), both showing prominent symptoms during the infantile stage.

In a study published in 2025, Trujillano et al. [11] reported that feeding difficulties (90.5%), hypotonia (85.7%), and GERD (82.4%) were the most frequently observed features. This study represents the first comprehensive characterization of a Spanish BRPS cohort and provides the most extensive phenotypic assessment reported to date. Both of our patients exhibited feeding difficulties and hypotonia, which were among the most frequently reported clinical features in the largest phenotypic characterization of BRPS published to date.

Although dysmorphic facial features varied among patients, characteristic facial features have been reported in approximately 93% of the cases. Both of our patients shared common physical features, including hypertelorism and a prominent forehead. In previous studies, no consistent or characteristic abnormalities were observed on brain MRI. Brain imaging was normal in most individuals; however, some showed nonspecific abnormalities, including white matter loss [3]. In the study by Balasubramanian et al. [9], brain MRI only showed nonspecific features with white matter changes (3/12) and vermis hypoplasia (1/12). Cranial MRI of our first patient showed a diffuse and symmetric increase in white matter signal intensity, a finding that correlates with previously described white matter abnormalities in the literature.

The 5‐year‐old Japanese girl reported by Hori et al. [12] carried the same splice‐site variant in the *ASXL3* gene (c.3047+1G > *A*) that was identified in our first patient. This patient had feeding difficulties, developmental delay, and by the age of 4 years, she could speak only a few words. Developmental milestones of our 2‐year‐old patient were also delayed and speech has not developed. Both patients showed failure to thrive. Japanese girl had a prominent forehead, microcephaly, and depressed nasal ridge, which are similar to our patient.

To our knowledge, the *ASXL3* c.1864_1867dup (p.Thr623Metfs25) variant identified in the second patient has not been previously reported in databases such as PubMed or ClinVar and is, therefore, classified as a novel variant. *ASXL3* variants have predominantly been detected in exons 11 and 12, which together account for approximately 83% of the total coding sequence of the *ASXL3* gene, likely reflecting their considerable size. The variant identified in the first patient was located at the splice donor site 3′ of exon 11, and the variant identified in the second patient was also located within exon 11. As the variant identified in the second patient is considered to be novel, its genotype–phenotype correlation can be clarified in the future through the identification of similar variants.

## 5. Conclusion

Global developmental delay is one of the most common reasons for referral to genetic clinics in the pediatric population. BRPS is associated with a nonspecific neurodevelopmental phenotype, and no definitive diagnostic clinical criteria have been established to date. At present, next‐generation sequencing enables the diagnosis of numerous genetic disorders in patients for whom no specific syndromic diagnosis can be made based on clinical features alone. Our study aimed to support the clinical characteristics of this rarely reported variant in the literature and to contribute to the literature by presenting the clinical features of a variant considered to be novel.

## Funding

No funding was received for this manuscript.

## Conflicts of Interest

The authors declare no conflicts of interest.

## Data Availability

The data that support the findings of this study are available on request from the corresponding author. The data are not publicly available due to privacy or ethical restrictions.
